# Establishing a new periodontitis-like intrabony maxillary defect in rats for investigation on bone regeneration

**DOI:** 10.1038/s41598-025-27201-8

**Published:** 2025-11-10

**Authors:** Marius Heitzer, Philipp Winnand, Mark Ooms, Kristian Kniha, Zuzanna Magnuska, Fabian Kiessling, Frank Hölzle, Ali Modabber

**Affiliations:** 1 Department of Oral and Maxillofacial Surgery, University Hospital of RWTH Aachen, Pauwelsstraße 30, 52074 Aachen, Germany; 2 Institute for Experimental Molecular Imaging, RWTH Aachen University, Forckenbeckstraße 55, Aachen, Germany

**Keywords:** Preclinical research, Imaging and sensing

## Abstract

The high incidence rate of periodontal bone defects and the unique regeneration characteristics of periodontal bone require a specially designed animal jawbone defect model to evaluate the appropriate periodontal bone regeneration procedure. The aim of the present investigation was to develop a reproducible, quantifiability and easy to implement periodontitis-like intrabony maxillary defect model in rats that allows investigation on bone regeneration. Ten upper jaws of rats were analyzed by micro-CT (µCT) imaging according to the bone dimensions for an appropriate position of a three-walled bone defect. A total of 30 intrabony defects measuring 1 × 1 × 1 mm were created using a split-mouth model on the palatal side of the maxillary first molar using ultrasonic surgery. 6 bone defects served as control. 20 bone defects were filled with alloplastic and xenogeneic particulate bone graft, and µCT scans were performed to verify bone regeneration of the periodontitis-like three-walled bone defect after 12 weeks. After 12 weeks, the µCT examinations showed sufficient bone regeneration of the artificially created periodontitis-typical defects. The µCT images revealed no morphological differences between xenogeneic and alloplastic bone substitute material. No restrictions for the animals, dehiscences or wound healing disorders were evident during the entire study period. The presented minimally invasive rat model with bilateral periodontitis-typical intrabony defects palatal to the first upper molar represents a favorable model for the investigation of regenerative osseous processes within a small defect.

## Introduction

Jawbones are part of a multifunctional organ with considerable biological and mechanical activity^1,2^. Therefore, defects of the jawbone can be associated with severe dysfunction of the oral skeletal system, can lead to significant facial deformities^3^, resulting in a great clinical need for the reconstruction and regeneration of missing or lost jawbone^3^. Tooth loss, periodontitis and apical cysts occur with a high incidence and are the most common reasons for the development of a burr hole bone defect in the maxillofacial region^4^. Various preclinical approaches to the restoration of maxillofacial drill hole bone defects in the form of bone regenerative approaches are described in the literature^5,6^.

The use of autologous bone grafting material is currently considered the gold standard for the reconstruction of jawbone defects. Limitations of autologous bone grafts include additional donor site morbidities and limited availability of bone material^7^. Therefore, there is a rigorous search for alternatives to autologous bone grafts to avoid invasiveness at the donor site in the treatment of jawbone defects^8,9^. In the search for alternative materials, alloplastic, allogenic, and xenogeneic bone grafts are being investigated in numerous studies with the aim of achieving bone regeneration^8,10–12^. Currently, animal experiments are an indispensable way of evaluating new regenerative treatments in order to shed light into the complex bone healing processes within a living organism. This suggests that a defect model should be established to study regenerative therapies in the jawbone.

Although some animal models for bone defects have been developed in long bones or cranial bones, the jawbones have unique tissue development origins and different regenerative environments compared to other bones^4^. Therefore, various animal models for the regeneration of bone defects in the jaw region have already been described in the literature^4,11–13^. A frequently used model for bone defects in the jaw region in small animals consists of a circular, penetrating defect, which is surgically created extra orally and is in the region of the mandibular ramus^4,13^. In addition to the limitation that bone regenerative processes are examined outside the oral cavity, the risk to the animals of injury to important structures through the extraoral approach must be regarded as disadvantageous^4^.

Other models for studying bone regeneration in rats include more traumatic procedures in the form of penetrating or segmental defects^11,14^. Structural support is required to bridge the non-contiguous gap of the interrupted bone.

Animal models with burr hole defects offer the advantage that only one side of the bone is injured and the remaining bone tissue surrounds the defect; this is particularly favorable for the investigation of bone regeneration materials in the form of particles or powders^4^. In addition, intrabony defects usually occur near the teeth, where the regeneration environment is mechanically dynamic and caused by chewing movements^6^. In order to create a model with the most realistic situation possible, the intrabony defect must preferably be directly adjacent to a tooth in order to be able to reproduce the movements of the teeth and the influence of the mechanical dynamics on the process of bone modeling. Existing models that meet these requirements are generated by bone defects at the mesial root of the first mandibular molar or maxillary molar in rats^15,16^. However, the limitations of these defect models are the near absence of an anterior defect wall. A concave defect morphology, on the other hand, is considered a favorable prerequisite for the feasibility of vertical augmentation of a bone defect using bone substitute materials^17^. Therefore, these surgically created small defects at the mesial root in the rat model are not ideal for the use of granulated bone substitute material but rather for the investigation of liquid substances and their periodontal regeneration potential.

The present investigation introduces a new periodontitis-like intrabony maxillary defect in rats that enables investigations of bone regeneration directly adjacent to a tooth. By creating standardized maxillary defects, this model enables realistic investigations of bone regeneration processes in mechanically loaded bone and, on the other hand, the artificial bone defect offers a reproducible, minimally invasive and yet sufficiently dimensioned defect size with a suitable defect morphology for the insertion of bone replacement materials.

## Materials and methods

### Animal welfare

Adult male Sprague-Dawley rats (250–300 g bodyweight) represent an established rodent model for studies on jawbone regeneration^4,18^, and were purchased for this study (Janvier Labs, Le Genest-Saint-Isle, France). All methods of this study are reported in accordance with the ARRIVE guidelines^19^, and were approved by the Governmental Animal Care and Use Committee of North Rhine-Westphalia (AZ 81-02.04.2020A458). Additionally, all procedures were carried out in accordance to the German animal protection law and the EU Directive 2010/63. Before the start of the experiment, the animals were kept in quarantine, and housed in cages for 7 days for acclimatization. The animals were housed in a pathogen-free environment under a 12-h light/12-h dark cycle with food, and water ad libitum. The animals were provided with softened food for 7 days postoperatively to prevent irritation of the wounds.

### Sample size calculation

The existing literature on animal studies about intraoral jawbone regeneration was reviewed to calculate a suitable sample size. As there is no comparable published data on bone regeneration with the animal model we described, the authors relied on established bone defect models and comparable animal studies^4,15,16^. In these studies, the sample sizes ranged from 5 to 12 animals for the evaluation of the different bone healing therapies. Based on these references, and assuming a significance level of α = 0.05 and a statistical power of 95%, a large effect size (d = 0.8) was determined according to Cohen’s d guidelines. Accordingly, the sample size in the present study was calculated based on an alpha level of 0.05, an effect size of 0.8, and a power of 95% using the statistical program G* Power Version 3.1.9.6 (Heinrich-Heine-Universität, Düsseldorf, Germany). Based on these parameters, a sample size of 8 bone defects was deemed appropriate. To ensure robustness, at least 10 bone defects per bone graft group were included in the present study. Additionally, six bone defects without allogeneic or xenogeneic bone graft served as controls.

### Micro computed tomography (µCT) of bone defects

To evaluate a suitable defect localization in the upper jaw, 3 animals were imaged before the start of the experiment. In the trial, the 15 animals were imaged immediately after the operation and 12 weeks after the procedure. In vivo imaging was conducted by the use of the micro-computed tomography (µCT) system (U-CT OI, MILabs, Utrecht, Netherlands). In vivo imaging before surgery and 12 weeks after operation were carried out under general inhalation anesthesia by isoflurane (induction with 5 vol% isoflurane + 5 L O2/min; maintenance with 2 vol% isoflurane + 2 L O2/min) (Abbott GmbH & Co. KG, Wiesbaden, Germany). The µCT scans of rat heads were performed within a field of view of 88 × 88 × 230 mm (x, y, z-axis), at a voltage of 65 kV, a current of 0.13 mA and an exposure time of 300 ms. The images were obtained by ultra-focus magnification through 360° rotation at 0.75° increments with 0.3 s/degree. µCT data were reconstructed at a 40 μm isotropic voxel size. Preoperative images were analysed to identify the best location for a three-walled defect of the upper jaw.

**Fig. 1:**
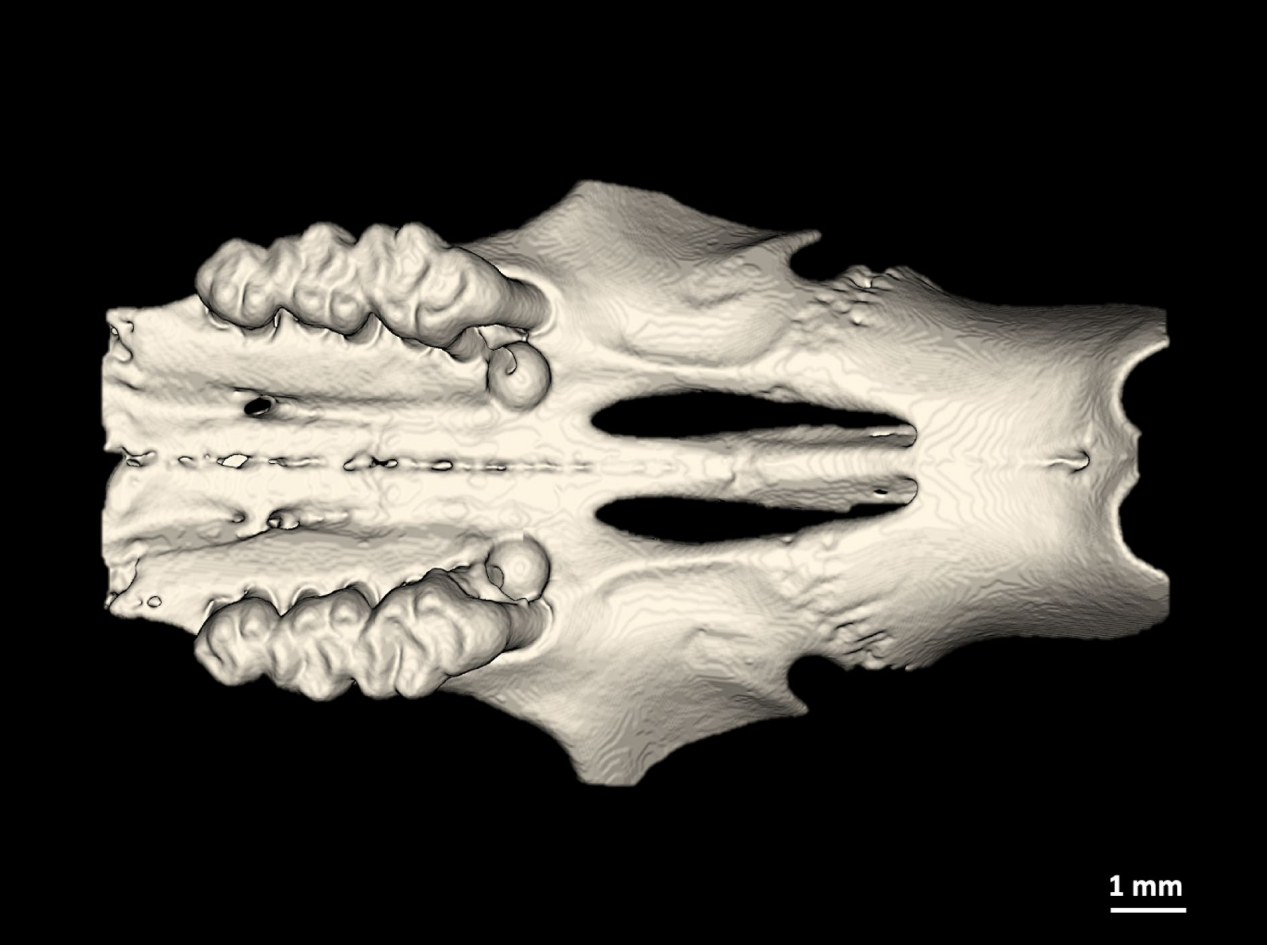
Postoperative visualization of a rat maxilla with created intrabony defects on both first upper molar without bone graft material reconstructed from µCT images.

### Surgical procedure

**Fig. 2:**
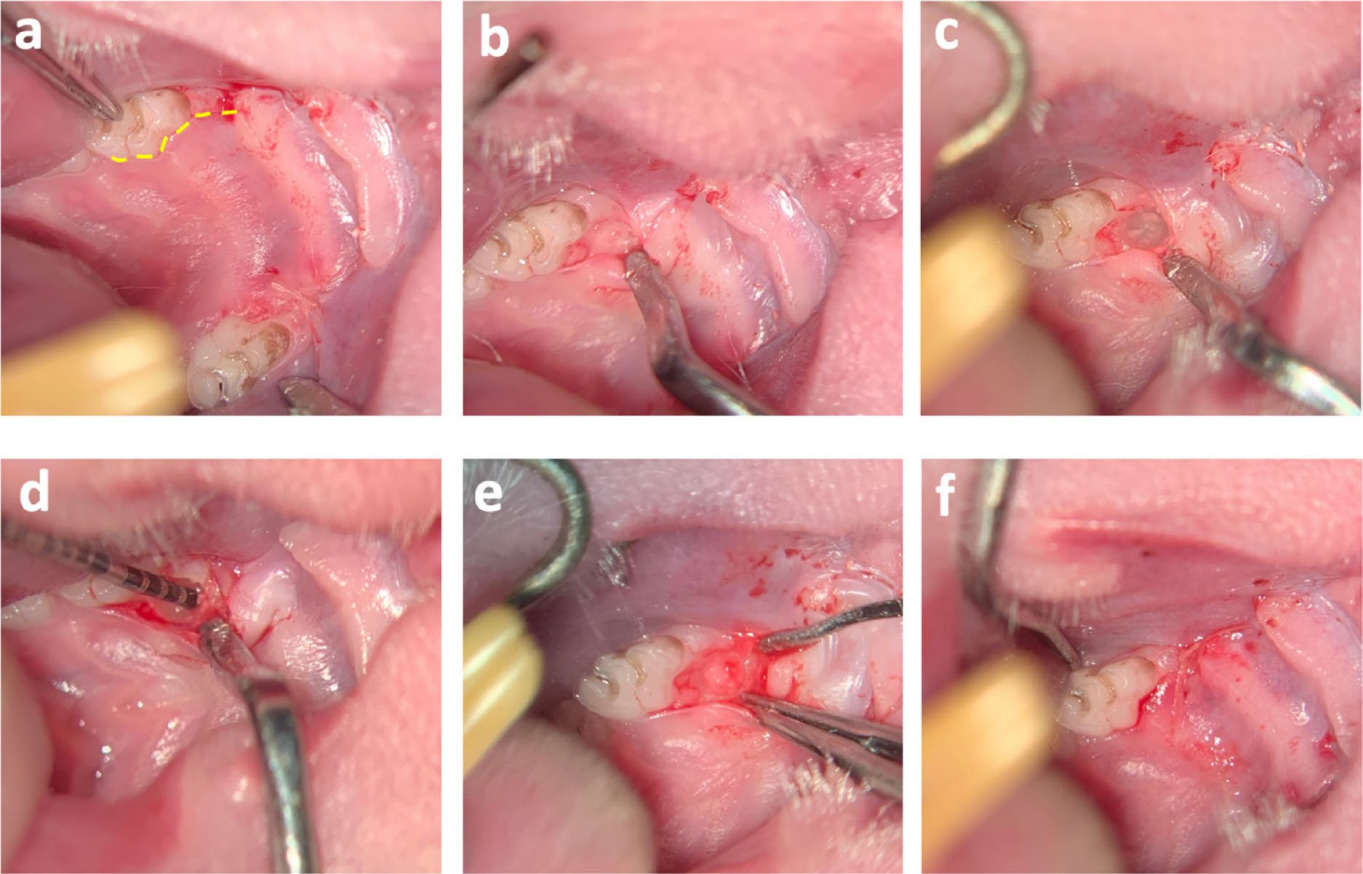
Photographic documentation of the individual surgical steps. **a** Representation of the incision, which is also illustrated by the yellow lines. The opposite side of the jaw has already been operated on the first molar and closed with sutures. **b** Blunt subperiosteal preparation and formation of a mucoperiosteal flap. **c** The artificially created three-walled intrabony defect. **d** Monitoring the defect size using a periodontal probe; **e** particulate bone substitute material inserted into the defect; **f** postoperative situation with tension-free adaptation of the wound edges and gingival suture.

All operation were performed under microscopic magnification (OPMI pico f170, Carl Zeiss AG, Oberkochen, Germany). The analgesic buprenorphin (0.05 mg/kg bodyweight) was injected subcutaneously 30 min before operation. Intraperitoneal injection of sodium pentobarbital (30–50 mg/kg bodyweight) served as narcotic. The animals were placed upside down in the supine position with a slight angulation so that no further aspiration protection for fluids or blood was required. To relieve postoperative pain and intraoperative bleeding, additionally local anesthesia using articainhydrochlorid 4% was injected submucosally. A mucoperiosteal flap was developed palatally through a palatal marginal incision on the first upper molar with mesial incision. The mucoperiosteal flap was gently removed and an intrabony defect between the mesial and the first palatal root with a diameter of 1 mm long, 1 mm wide, and 1 mm deep was created using piezosurgery instruments (OP5A, Mectron, Germany, Ref. 03380012) with a diameter of 0.5 mm and a diamond grain size of 90 μm. The dimensions of the bone defect was constantly monitored using a clinical periodontal probe until the required size was obtained (W × L × D; 1 × 1 × 1 mm). This procedure was performed analogously on the first maxillary molar on the opposite side of the jaw (Fig. 1). In a total of 15 animals, 30 bone defects were created on both sides of the maxilla. Of these, 10 intrabony defects were filled with an alloplastic bone graft (maxresorb^®^, botiss biomaterials GmbH, Germany), 10 intrabony defects were filled with a particulate xenogenic bone graft substitute (cerabone^®^, botiss biomaterials GmbH, Germany) and 10 intrabony defects served as controls. At the end, the mucoperiosteal flap was placed back in its original position and sutured tension-free with single 7-0 button sutures (Vicryl 7-0, Ethicon Inc., USA) in the area of the mesial incision. Postoperative controls and inspections of the oral cavity were carried out in accordance with the approved regulations of the animal protocol (Fig. 2).

### Statistical analysis

Data were analyzed using the GraphPad Prism 10.1.1 program (GraphPad Software, Inc., San Diego, CA, USA). Normal distribution was tested using the D`Agostino & Pearson test and the Shapiro–Wilk test. The parametric data were analyzed using ANOVA and Tukey´s multiple comparison test. The presented data in this paper are shown as means ± standard deviation (SD). Differences between the groups were considered statistically significant at *p* ≤ 0.05.

## Results

An adequate bone defect volume is vital for evaluating bone regeneration strategies, particularly in small animals, which typically possess very limited bone volume. Therefore, preoperative images were analysed to identify the location in the maxilla of rats that meets the requirements in the immediate vicinity of a tooth, has sufficient bone availability for the creation of a three-walled defect and at the same time can generate an animal-friendly and minimally invasive procedure in relation to important adjacent structures. A total of 10 upper jaws of rats were analysed and measured for this purpose. The anterior sections of the maxilla contain the two incisors and the bone there contains the dorsal roots of the incisors. In the direct neighborhood of the nasal tube, the bone supply here was very low. The major feature of the medial maxilla area is lack of teeth and a very thin bone lamella due to the neighboring nasal tube. In the distal area of the molars, the bone volume was also too small to create a bone defect of sufficient size. Anterior to the first maxillary molar, the bone thickness increased significantly in the vertical direction towards the first molar and showed sufficient bone availability in the area between the mesial and mesiopalatal root to create a bone defect measuring 1 × 1 × 1 mm in the immediate vicinity of a tooth. The bone structures that were determined in advance and measured palatal of the first upper molar are shown in Table 1.

**Table 1:** The bone thicknesses determination of investigation by µCT. Measurements were conducted palatial of the first upper molar at the position where the intrabony defects were created. Numbers are indicated as means and standard deviations.

	Buccolingual	Mesiodistal	Max. craniocaudal	Min. craniocaudal
Dimensions (mm)	2.07 ± 0.14	2.05 ± 0.16	2.98 ± 0.19	1.76 ± 0.06

**Fig. 3:**
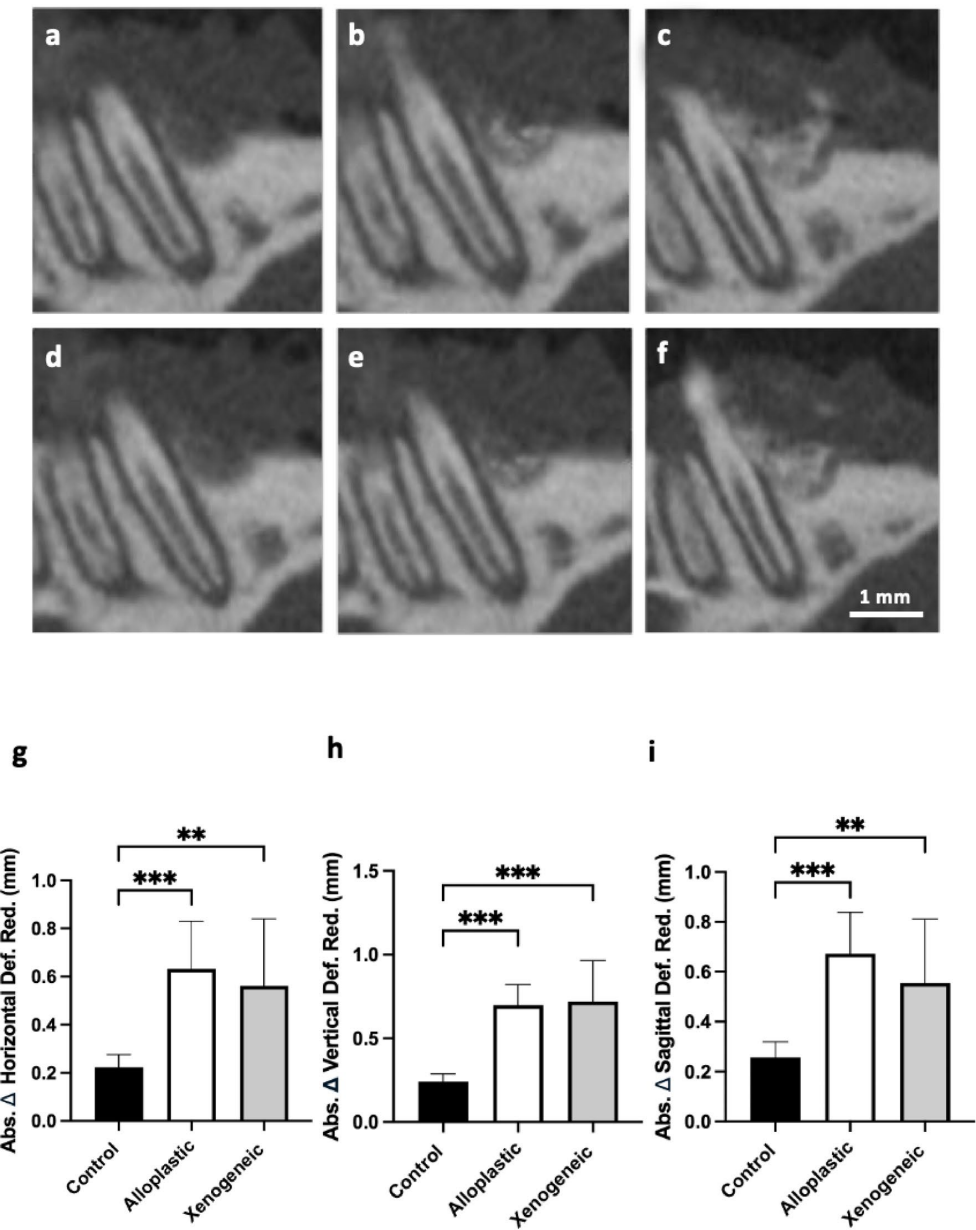
µCT images of intrabony defect in the sagittal plane with **a** control defect, **b** alloplastic and **c** xenogeneic bone graft after operation. Intrabony defect in the sagittal plane with **d** control defect, **e** alloplastic and **f** xenogeneic bone graft after 12 weeks. Graphical representation of absolute difference in bone reduction in **g** horizontal (**p = 0.002; ***p ≤ 0.001), **h** vertical (***p ≤ 0.001), and **i** sagittal plane (**p = 0.003; ***p ≤ 0.001).

Postoperatively, the inserted bone graft material could be easily separated from the original bone. This was regularly located in the inserted three-walled defect. After 12 weeks, the reduction of the the intrabony defects in the control group was 0.22 ± 0.05 mm in the horizontal plane, 0.24 ± 0.05 mm in the vertical plane and 0.26 ± 0.06 mm in the sagittal plane. No significant reduction in the size of the bone defects was observed. In contrast to the control group, the reduction of the bone defects with alloplastic bone graft were significantly smaller in the horizontal plane with 0.63 ± 0.19 mm (*p* ≤ 0.001), in the vertical plane 0.69 ± 0.12 mm (*p* ≤ 0.001) and in the sagittal plane 0.67 ± 0.17 mm (*p* ≤ 0.001). Similarly, compared to the control group the size of the bone defects with xenogeneic bone graft substitute material was significantly reduced in the same 12-week period with 0.56 ± 0.28 mm (*p* = 0.002) in the horizontal plane, 0.72 ± 0.24 mm (*p* ≤ 0.001) in the vertical plane and 0.56 ± 0. 25 mm (*p* = 0.003) in the sagittal plane (Table 2; Fig. 3). During the entire observation period, no signs of intraoral dehiscence were observed at the first upper molar.

**Table 2:** Evaluation of absolute differences of defect sizes by µCT. Measurements of defect size were conducted in the horizontal, vertical and sagittal plane. Numbers are indicated as means and standard deviations.

Abs. Δ defect reduction (mm)	Horizontal	Vertical	Sagittal
Control	0.22 ± 0.05	0.24 ± 0.05	0.26 ± 0.06
Alloplastic	0.63 ± 0.19	0.69 ± 0.12	0.67 ± 0.17
Xenogeneix	0.56 ± 0.28	0.72 ± 0.24	0.56 ± 0. 25

## Discussion

The loss of jawbone is a widespread therapeutic issue in oral and maxillofacial surgery in the rehabilitation of affected jaws^3^. The high incidence rate of periodontitis and tooth loss means that ridge-associated burr hole bone defects in the immediate vicinity of teeth are the most common form of bone loss in jaws^4^. The great therapeutic potential of various regenerative approaches using different bone grafts for bony regeneration of jawbone defects has already been extensively described in the literature^4–6,10,11,13,20^. Nevertheless, the investigation on the regeneration of these bone defects is carried out in animal models, which are not able to adequately represent the mechanical, biological and oral bone remodeling conditions under the influence of the stomatognathic system^4–6,13^. The emerging number of novel stem cell-based or exosome-guided strategies for bone regeneration creates an urgent need to investigate the knowledge gained in realistic animal models^10,13,20,21^.

In this study, we focused on the development of a novel small animal model that has a reproducible periodontitis-like intrabony maxillary bone defect and provides realistic conditions for investigations on bone regeneration. These realistic conditions include the mechanical loading of the alveolar ridge by teeth, which has a partially stimulating influence on the bone remodeling. In addition, an animal-friendly and minimally invasive model was to be developed, as well as a model with a sufficient defect size for the insertion of particulate bone substitute material.

Animal models with periodontitis-associated bone resorption are widely described in the literature^22–24^. With 72.7% the procedure with induced periodontitis, in which bone resorption can be observed after a few weeks using a circular ligature around the tooth, is an often-used small animal model^22–24^. Significant disadvantages of this model are the varying degrees of bone resorption in the course of induced periodontitis. Gao et al. therefore describe an optimized ligature/bone defect-induced periodontitis model in which vestibular bone defects with a diameter of 1 mm wide, 1 mm long, and 1 mm deep were created after surgical defect preparation between the first and second upper molar and a 5 − 0 ligature was applied^23^. A comparable study by Cao et al. used an intrabony defect model of periodontitis to investigate the bone healing of a vertical, 1.5 mm deep, surgically created bone defect in the maxilla^25^. In a control group without periodontitis, spontaneous bone healing of the defect of around 0.59 ± 0.12 mm in the vertical dimension was described after about 6 weeks, which corresponds to the slower bone healing of the control group in our experiment. Analogous to these defect sizes, we have developed a bone defect model in our study that has comparable defect sizes. The vertical defect reduction was 0.24 ± 0.05 mm with remaining defect dimensions of the control group in our study with 0.74 ± 0.1 mm after 12 weeks and, in contrast to animal models with a burr hole defect, showed a certain degree of spontaneous healing. However, in contrast to the intrabony defects treated with alloplastic or xenogeneic bone graft substitutes, the defects in the control group showed less regeneration and bone healing. The fundamental difference in the model presented by us is the complete hard tissue boundary of the defect, which is a basic prerequisite for research into bone regenerative processes. Due to the wedge-shaped design of the vestibular open defect described by Gao et al.^23^ and the combined defect of ligation and surgical periodontitis defect preparation described by Cao et al.^25^ these rodent models represent favorable circumstances for the investigation of periodontal therapies in an inflammatory milieu in contact and seem less suitable for the evaluation of regenerative therapies using bone substitutes in the form of a pure bone regeneration study.

As there is a lack of animal studies in the literature with comparable bone defects treated with the same bone substitutes, the observed bone regeneration in the presence of the bone substitutes can only be compared with existing data to a limited extent. In general, the use of bone substitutes can promote bone healing of bone defects and reduce the resorption of the alveolar ridge^26^. Accordingly, the bone defects in our bone defect model with alloplastic and xenogeneic bone graft material showed significant bone regeneration in the vertical dimensions with an absolute defect reduction of 0.69 ± 0.12 mm with the alloplastic bone graft material and 0.72 ± 0.24 mm with the xenogeneic bone graft material, respectively.

The small size of the intrabony defect is a general limitation of a minimally invasive procedure. In contrast to critical-size defects, in which bone healing can only take place through the insertion of bone replacement materials^4,5,27^, smaller defects are subject to a certain degree of spontaneous healing^25^. As described in literature, this spontaneous healing of bone defects is inferior to the use of bone substitutes in terms of higher atrophy of the alveolar ridge^26^. In the jawbone of a rodent model, which should have an intrabony defect within the oral cavity and in the vicinity of teeth, the realization of a burr hole defect is not feasible due to the required dimensions of at least 6 × 2 mm for the absence of spontaneous bone healing^4^. Although the use of large animal models can help to overcome these limitations, it is still advisable to perform small animal studies first to allow initial screening and exclude material candidates with suboptimal performance^4^. This approach is in line with the 3R principles^28^, which advocate replacing large animals when small animal models are sufficient to achieve the research objectives. Thus, larger scaffolds^5,6^, which require a larger defect for implantation, cannot be investigated in this model with limited bone volume, especially in the maxillofacial bone area, which is a common limitation of rodent models^4^. On the other hand, the model seems to be suitable for the investigation of novel cellular regenerative procedures with bone substitutes^8,13^.

The use of piezosurgery allows for good control during creation of bone defects^11^. Nevertheless, many studies use rotating or drilling instruments for the creation of bone defects^4,5,23,29^. In this study, the authors deliberately decided to use a predictable and accurate procedure for a reliable maxillary defect model. This implies that the defect is directly adjacent to a tooth. The use of piezosurgery not only offers the advantage of good controllability, but also has the considerable advantage that the structures of the neighboring teeth can be spared. Moreover, Esteves et al.^29^ reported of higher amount of newly formed bone observed in tibial defects of rats by piezosurgery compared to conventional drilling. For this reason, it has proven successful to use more gentle ablative procedures with piezosurgery for the artificial creation of bone defects close to a tooth^15,30^.

Reproducibility not only enables favorable comparability of therapies, but also often represents a significant limitation in bone remodeling investigations^23^. The reproducibility of the defect dimensions also enables a safe prognosis and reliability of the surgical result. Especially in the context of animal welfare, reproducible and predictable operations in the maxilla of rodents are of utmost importance. Since rodents are obligatorily redundant in nasal inhalation, the integrity of the nasal tube and nasal mucosa is crucial^11^. To avoid this, the defect in the maxilla in our model was deliberately chosen at a site with sufficient bone dimensioning towards the nose. Exact reproducibility not only enables good comparability of the treatments, but also a reliably predictable surgical result. Accuracy is of particular importance in maxillary surgery in rats, especially against the background of animal welfare.

At present, there is a lack of animal models that include a bone defect model with consideration of mechanical tooth loading of the alveolar ridge and its influence on bone regeneration. The minimally invasive procedure presented in this manuscript proved to be a favorable procedure. Animal models with burr hole defects in the mandibular angle or mandibular corpus require extraoral access and are associated with increased comorbidities and potential damage to important structures^4^. Although these models remote from teeth offer the advantage of a more sterile procedure, they do not represent realistic stomatognathic conditions as they are not in contact with teeth.

Existing rat models with intrabony periodontal defects in direct location on a tooth, on the other hand, offer the possibility of more realistic in vivo studies of bone regenerative processes in the jaw. These models are artificially created either at the mesial root of the lower first molar or at the mesial root of the upper first molar^15,16^. In these models, due to the flat defect design regenerative processes are predominantly investigated using cellular, liquid or injectable substances^15,16^. Although these three-walled defect models represent the mechanical influence of teeth, they have considerable limitations in terms of insufficient defect walls and unfavorable defect design due to the anatomical conditions, making these bone defects unsuitable for research into particulate or solid bone substitutes. To the authors’ current knowledge, this manuscript is the first to describe the use of an intrabony periodontitis-typical defect on the palatal side of the maxillary first molar, between the mesial and first palatal root, for in vivo µCT studies of osseous regeneration using particulate bone grafting material. The collected µCT examinations indicate a reduction of the intrabony maxillary defect over a period of 12 weeks, which proves that this model meets the requirements of a favorable, minimally invasive procedure and is suitable for osseous regeneration investigations.

The model described in this manuscript is able to investigate the evaluation of bone regeneration in a realistic bone defect of the maxilla. Nevertheless, there are limitations in almost every small animal model described for studying bone regeneration^4,31^. One limitation of our defect model is based on the presence of the thin bone lamella. Therefore, the roots of the first molars do not have direct and continuous contact with the bone defects, so the deficiency of the model is the lack of root cement for the investigation of periodontal regeneration. Thus, the model as described is only able to depict the bone regeneration of a bone defect and not the regeneration of a periodontal defect. On the other hand, the model has the advantage that it can be easily and flexibly adapted for the examination of periodontal regeneration of a three-walled intrabony defect by removing the bone lamella at the tooth root and gently smoothing the root surface. In consideration of the described limitation that the defect is a strictly bone-only defect, the authors prefer to describe the presented model as a *periodontitis-like intrabony maxillary bone defect*.

Histological techniques are an important examination method for the microscopic and morphological assessment of bone tissue. On the other hand, this technique is subject to considerable limitations, which are primarily due to its labor-intensive and time-consuming nature. Additionally, these methods can compromise sample integrity during histological processing, and repeated measurements on the same specimen over time are not feasible^32^. To overcome these challenges, various three-dimensional (3D) imaging techniques are increasingly employed. Among them, µCT has become a widely accepted method for evaluating mineralized bone structures, owing to its high reproducibility and measurement accuracy, as demonstrated in several studies. µCT is particularly well-suited for in vivo monitoring of bone regeneration processes^32–34^. Furthermore, µCT is an established modality for microtomographic evaluation of bone remodelling and bone regeneration^32,34^. Comparative studies between traditional two-dimensional histology and 3D µCT have shown that µCT enables non-destructive, rapid, and precise radiological assessment of small bone specimens^32^. Furthermore, radiographic assessment is the tool of choice for analyzing bone regeneration with bone graft materials in clinical studies and enables an established and accurate examination of bone defects^26^. Against the background of the 3R strategies (refinement, reduction, and replacement) according to Burden et al. ^28^, an in vivo µCT examination allows a detailed structural analysis without invasive biopsies^35^ or killing of animals.

## Conclusion

By analyzing the anatomic structures of rat maxilla in µCT images, comparing the thickness of the upper jaw and investigation on bone dimensions of various parts of the maxilla, identifying the critical landmarks of the maxilla, and investigations on the regenerative capacity, we successfully developed a standardized and reproduceable intrabony defects in rodents. Further investigations on bone regeneration using the new defect model are needed to verify our findings.

## Data Availability

All data generated for this study are available from the corresponding authors upon reasonable request.
